# Arterial stiffness in severe aortic stenosis following Transcatheter Aortic Valve Implantation (TAVI) compared to Surgical Aortic Valve Replacement (SAVR)

**DOI:** 10.1186/1532-429X-16-S1-O34

**Published:** 2014-01-16

**Authors:** Tarique A Musa, Akhlaque Uddin, Timothy A Fairbairn, Christopher D Steadman, Ananth Kidambi, Manish Motwani, David P Ripley, Adam K McDiarmid, Peter P Swoboda, Steven Sourbron, Sven Plein, Gerry P McCann, John P Greenwood

**Affiliations:** 1 MCRC & LIGHT, University of Leeds, Leeds, South Yorkshire, UK; 2 Cardiovascular Sciences, National Institute of Health Research, Leicester Cardiovascular Biomedical Research Unit, Leicester, UK

## Background

Arterial stiffness is an independent predictor of cardiovascular mortality and can be assessed by CMR measurement of aortic distensibility (AD) and pulse wave velocity (PWV). We sought to determine AD and PWV in patients with severe symptomatic aortic stenosis and identify changes following TAVI compared to SAVR.

## Methods

**Figure 1:**
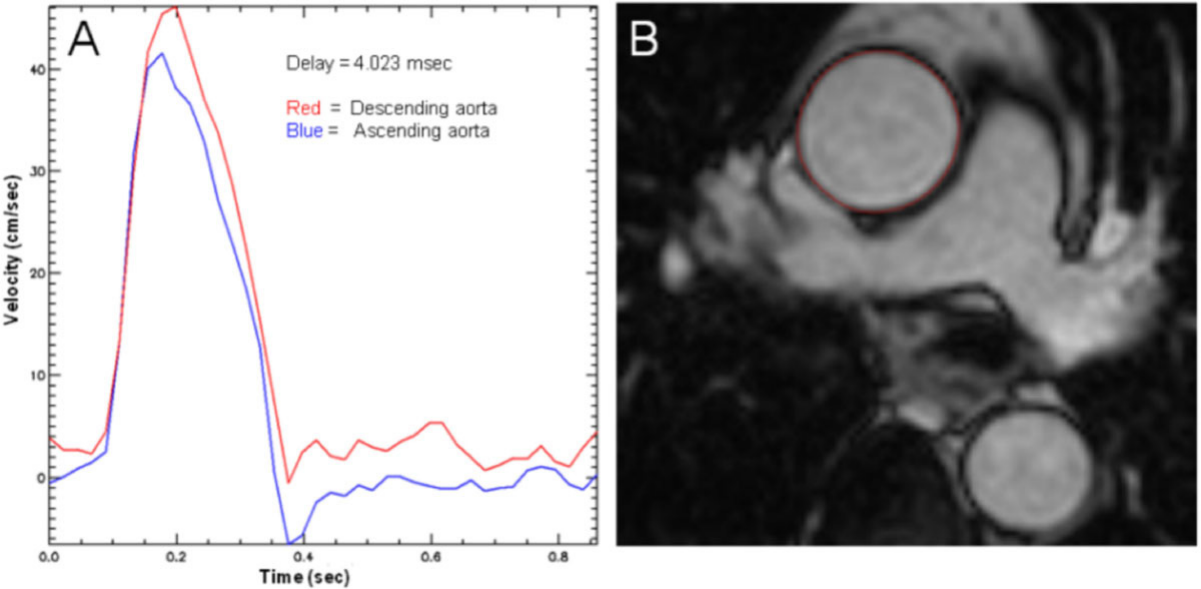
**A) Time-Velocity curves derived using PMI software to calculate foot-foot delay (curve in ascending aorta is shifted over the measured delay as a visual verification of the result) B) Aortic cross sectional measurements made by manual planimetry (red contour) of the ascending aortic endovascular-blood pool interface at minimal and maximal distension**.

Forty-eight patients underwent identical paired 1.5T CMR scans (Intera, Philips or Avanto, Siemens). Brachial artery blood pressure was recorded by Dinamap (Critikon, Tampa, Fl, USA). Multi-phase steady state free precession (SSFP) cine imaging (50 phases) was acquired in a axial plane to the thoracic aorta at the level of the pulmonary artery bifurcation (Ascending aorta (AA) and proximal descending aorta (PDA)). AD (mmHg-1) was calculated following the contouring of the aortic region of interest, Figure 1A (QMass V7.5, Medis, The Netherlands), using the equation; (Aortic max lumen area-Aortic min lumen area)/(Aortic min lumen area × [Systolic BP - Diastolic BP]). Aortic PWV was assessed using identical planning with retrospectively gated, through plane, phase-contrast velocity encoded images (breath-hold, single slice, 10 mm thick, 40 phases, typical FOV 350, RFOV 85, VENC 200 cm/s). Offline analysis was performed using previously published algorithms implemented in the in-house software PMI 0.4. The velocity encoded images of the AA and PDA were manually contoured to derive velocity-time curves. The distance (mm) between AA and PDA was measured manually from in-plane saggital-oblique SSFP images of the aortic arch. PWV (m/s) was calculated using the transit-time method (foot-foot delay, Figure 1B).

## Results

27 SAVR patients (age 71.8 ± 7.0 years, 75% male, EuroSCORE II 1.43 ± 0.44%) and 21 TAVI patients (age 81.7 ± 6.3 years, 52% male, EuroSCORE II 6.32 ± 5.99%) were studied before and 6 months following valve replacement. Arterial pulse pressure significantly increased following SAVR (57 ± 19.6 vs. 63 ± 14.6 mmHg, p < 0.05) but not after TAVI (68 ± 24.0 vs. 67 ± 21.6 mmHg, p = 0.91). AD significantly decreased post SAVR (2.00 ± 1.57 vs. 1.39 ± 0.69 × 10-3 mmHg-1, p < 0.05) whereas there was no change observed in the TAVI group (1.68 ± 0.80 vs. 1.76 ± 0.85 × 10-3 mmHg-1, p = 0.74). PWV significantly increased post-SAVR (6.69 ± 5.12 vs. 12.13 ± 6.22 ms-1, p = 0.01) whereas there was no change observed in the TAVI group (9.91 ± 9.32 vs. 12.42 ± 9.24 ms-1, p = 0.23).

## Conclusions

In patients with severe aortic stenosis, SAVR but not TAVI is associated with a significant increase in PWV and decrease in AD at 6 months. This increase in aortic stiffness may be a consequence of the different techniques of valve replacement and has potential long-term implications on cardiovascular mortality.

## Funding

This study was part-funded by the British Heart Foundation (BHF) (PG/11/126/29321). GP McCann and CD Steadman have received support from the NIHR Leicester Cardiovascular BRU.

**Figure 2:**
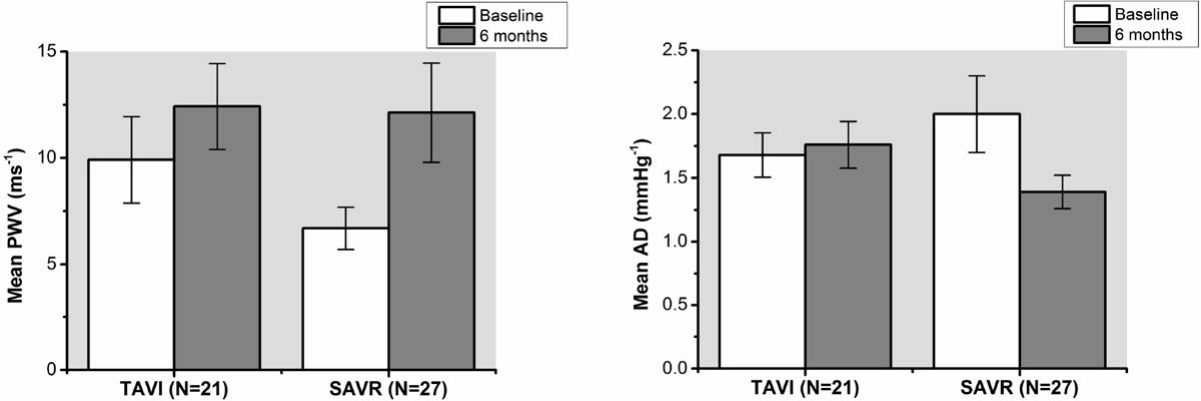
**Change in aortic distensibility and pulse wave velocity pre- and post-TAVI/SAVR**.

